# 1-{1-[(2-Chloro­thia­zol-5-yl)meth­yl]-5-methyl-1*H*-1,2,3-triazol-4-yl}ethanone

**DOI:** 10.1107/S1600536809042937

**Published:** 2009-10-23

**Authors:** Xiao-Bao Chen, Jia-Hua Tian, Jing Xu, Yong Yu, Qun Wang

**Affiliations:** a Institute of Medicinal Chemistry, Yunyang Medical College, Shiyan, 442000, People’s Republic of China

## Abstract

In the title compound, C_9_H_9_ClN_4_OS, the two rings enclose a dihedral angle of 84.67 (11)°. Inter­molecular C—H⋯O and C—H⋯N hydrogen bonds stabilize the crystal packing.

## Related literature

For the biological activity of triazole derivatives, see Najim *et al.* (2004 ▶); Liu *et al.* (2001 ▶). For the synthesis of the title compound, see: Chen & Shi (2008 ▶).
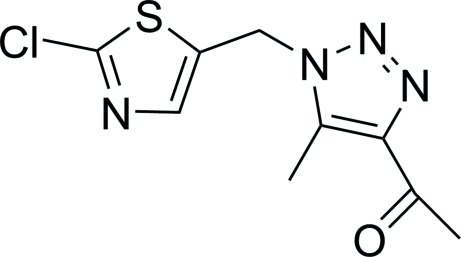


## Experimental

### 

#### Crystal data

C_9_H_9_ClN_4_OS
*M* *_r_* = 256.71
Orthorhombic, 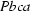

*a* = 10.5421 (6) Å
*b* = 11.1494 (6) Å
*c* = 19.8557 (10) Å
*V* = 2333.8 (2) Å^3^
*Z* = 8
Mo *K*α radiation
μ = 0.49 mm^−1^
*T* = 298 K
0.16 × 0.10 × 0.10 mm

#### Data collection

Bruker SMART APEX CCD area-detector diffractometer
Absorption correction: none
22708 measured reflections
2556 independent reflections
2336 reflections with *I* > 2σ(*I*)
*R*_int_ = 0.046

#### Refinement

*R*[*F* ^2^ > 2σ(*F* ^2^)] = 0.047
*wR*(*F* ^2^) = 0.128
*S* = 1.15
2556 reflections
147 parameters
H-atom parameters constrained
Δρ_max_ = 0.29 e Å^−3^
Δρ_min_ = −0.19 e Å^−3^

### 

Data collection: *SMART* (Bruker, 2000 ▶); cell refinement: *SAINT* (Bruker, 2000 ▶); data reduction: *SAINT*; program(s) used to solve structure: *SHELXS97* (Sheldrick, 2008 ▶); program(s) used to refine structure: *SHELXL97* (Sheldrick, 2008 ▶); molecular graphics: *SHELXTL* (Sheldrick, 2008 ▶); software used to prepare material for publication: *SHELXTL*.

## Supplementary Material

Crystal structure: contains datablocks global, I. DOI: 10.1107/S1600536809042937/bt5103sup1.cif

Structure factors: contains datablocks I. DOI: 10.1107/S1600536809042937/bt5103Isup2.hkl

Additional supplementary materials:  crystallographic information; 3D view; checkCIF report

## Figures and Tables

**Table 1:** Hydrogen-bond geometry (Å, °)

*D*—H⋯*A*	*D*—H	H⋯*A*	*D*⋯*A*	*D*—H⋯*A*
C4—H4*B*⋯N3^i^	0.97	2.48	3.399 (3)	159
C4—H4*A*⋯O1^ii^	0.97	2.48	3.376 (3)	153
C7—H7*C*⋯O1^ii^	0.96	2.57	3.396 (3)	144

Symmetry codes: (i) 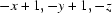; (ii) 
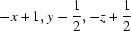
.

**Fig. 1.**
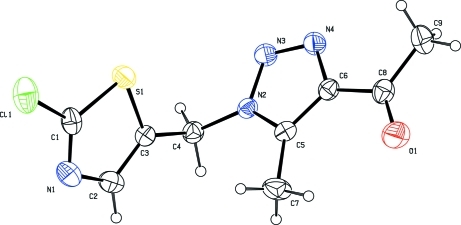
View of the molecular structure of the title compound, showing the atom labelling scheme. Displacement ellipsoids are drawn at the 50% probability level.

**Fig. 2.**
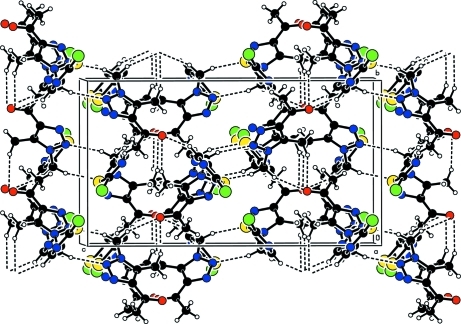
A partial view of the crystal packing of the title compound, showing the C—H···O and C—H···N hydrogen bonds as dashed lines.

## supplementary crystallographic information

### Comment

It is well known that many triazole-related molecules play an important role in the development of agrochemicals such as insecticides, nematocides, acaricide and plant growth regulators ( Najim *et al.*, 2004;       Liu *et al.*, 2001). The structure-activity relationship is very useful in the rational design of pharmaceuticals and agrochemicals. We report here the crystal structure of the title compound (Fig. 1), which was synthesized by adding a thiazole rings  to a 1,2,3-Triazole molecular framework. Intermolecular C—H···O and C—H···N hydrogen bonds contribute strongly to the stability of the crystal packing (Fig. 2).

### Experimental

Acetylacetone (2 mmol) and 5-azidomethyl-2-chlorothiazole (2 mmol) were added to a suspension of milled potassium carbonate (6 mmol) in DMSO (10 ml). The mixture was stirred at room temperature for 10 h (monitored by thin-layer chromatography) and poured to water (50 ml). The solid was collected by filtration, washed with water and diethyl ether, respectively, and dried to give 0.46 g of the title compound (yield 90%). Colorless crystals of (I) suitable for X-ray structure analysis were grown from acetone and petroleum ether (1:3, *v*/*v*).

### Refinement

H atoms bonded to C were placed at calculated positions, with C—H distances of 0.97 and 0.93Å for H atoms bonded to *sp*^3^ and *sp*^2^ C atoms, respectively. They were refined using a riding model, with *U*_iso_(H) = 1.2*U*_eq_(C) or 1.5*U*_eq_(methyl C). the methyl groups were allowed to rotate but not to tip.

### Crystal data

**Table:**

C_9_H_9_ClN_4_OS	*D*_x_ = 1.461 Mg m^−^^3^
*M**_r_* = 256.71	Mo *K*α radiation, λ = 0.71073 Å
Orthorhombic, *P**b**c**a*	Cell parameters from 9847 reflections
*a* = 10.5421 (6) Å	θ = 2.8–28.3°
*b* = 11.1494 (6) Å	µ = 0.49 mm^−^^1^
*c* = 19.8557 (10) Å	*T* = 298 K
*V* = 2333.8 (2) Å^3^	Block, colorless
*Z* = 8	0.16 × 0.10 × 0.10 mm
*F*(000) = 1056	

### Data collection

**Table:**

Bruker SMART APEX CCD area-detector diffractometer	2336 reflections with *I* > 2σ(*I*)
Radiation source: fine-focus sealed tube	*R*_int_ = 0.046
graphite	θ_max_ = 27.0°, θ_min_ = 2.1°
φ and ω scans	*h* = −13→13
22708 measured reflections	*k* = −14→14
2556 independent reflections	*l* = −25→25

### Refinement

**Table:**

Refinement on *F*^2^	Primary atom site location: structure-invariant direct methods
Least-squares matrix: full	Secondary atom site location: difference Fourier map
*R*[*F*^2^ > 2σ(*F*^2^)] = 0.047	Hydrogen site location: inferred from neighbouring sites
*wR*(*F*^2^) = 0.128	H-atom parameters constrained
*S* = 1.15	*w* = 1/[σ^2^(*F*_o_^2^) + (0.0593*P*)^2^ + 0.9336*P*] where *P* = (*F*_o_^2^ + 2*F*_c_^2^)/3
2556 reflections	(Δ/σ)_max_ = 0.002
147 parameters	Δρ_max_ = 0.29 e Å^−^^3^
0 restraints	Δρ_min_ = −0.19 e Å^−^^3^

### Special details

**Table:**

Geometry. All e.s.d.'s (except the e.s.d. in the dihedral angle between two l.s. planes) are estimated using the full covariance matrix. The cell e.s.d.'s are taken into account individually in the estimation of e.s.d.'s in distances, angles and torsion angles; correlations between e.s.d.'s in cell parameters are only used when they are defined by crystal symmetry. An approximate (isotropic) treatment of cell e.s.d.'s is used for estimating e.s.d.'s involving l.s. planes.
Refinement. Refinement of *F*^2^ against ALL reflections. The weighted *R*-factor *wR* and goodness of fit *S* are based on *F*^2^, conventional *R*-factors *R* are based on *F*, with *F* set to zero for negative *F*^2^. The threshold expression of *F*^2^ > σ(*F*^2^) is used only for calculating *R*-factors(gt) *etc*. and is not relevant to the choice of reflections for refinement. *R*-factors based on *F*^2^ are statistically about twice as large as those based on *F*, and *R*- factors based on ALL data will be even larger.

### Fractional atomic coordinates and isotropic or equivalent isotropic displacement parameters (Å^2^)

**Table:**

	*x*	*y*	*z*	*U*_iso_*/*U*_eq_	
C1	0.06077 (19)	0.5833 (2)	0.07494 (12)	0.0523 (5)	
C2	0.1710 (2)	0.4540 (2)	0.13106 (13)	0.0611 (6)	
H2	0.1838	0.3914	0.1613	0.073*	
C3	0.26783 (19)	0.50485 (18)	0.09799 (9)	0.0425 (4)	
C4	0.40502 (19)	0.47144 (17)	0.10133 (10)	0.0433 (4)	
H4A	0.4171	0.4137	0.1373	0.052*	
H4B	0.4291	0.4331	0.0594	0.052*	
C5	0.50309 (18)	0.64274 (16)	0.16817 (9)	0.0389 (4)	
C6	0.59612 (18)	0.72323 (17)	0.14998 (9)	0.0403 (4)	
C7	0.4283 (3)	0.6266 (2)	0.23107 (11)	0.0649 (7)	
H7A	0.3395	0.6336	0.2211	0.097*	
H7B	0.4519	0.6872	0.2631	0.097*	
H7C	0.4452	0.5488	0.2497	0.097*	
C8	0.6563 (2)	0.81698 (19)	0.19098 (11)	0.0492 (5)	
C9	0.7538 (2)	0.8946 (2)	0.15856 (13)	0.0637 (6)	
H9A	0.7830	0.9533	0.1903	0.096*	
H9B	0.7173	0.9344	0.1203	0.096*	
H9C	0.8239	0.8460	0.1441	0.096*	
Cl1	−0.06811 (6)	0.66155 (7)	0.04706 (5)	0.0803 (3)	
N1	0.05217 (18)	0.4984 (2)	0.11826 (11)	0.0659 (6)	
N2	0.48829 (14)	0.57468 (14)	0.11306 (7)	0.0380 (3)	
N3	0.56710 (16)	0.61037 (18)	0.06291 (9)	0.0499 (4)	
N4	0.63206 (16)	0.70047 (17)	0.08532 (8)	0.0498 (4)	
O1	0.6277 (2)	0.82866 (17)	0.24974 (9)	0.0745 (5)	
S1	0.21063 (5)	0.61575 (6)	0.04596 (3)	0.0553 (2)	

### Atomic displacement parameters (Å^2^)

**Table:**

	*U*^11^	*U*^22^	*U*^33^	*U*^12^	*U*^13^	*U*^23^
C1	0.0380 (10)	0.0567 (12)	0.0623 (13)	−0.0021 (9)	0.0002 (9)	−0.0114 (11)
C2	0.0553 (13)	0.0692 (15)	0.0589 (13)	−0.0073 (11)	0.0030 (10)	0.0170 (11)
C3	0.0443 (10)	0.0455 (10)	0.0376 (9)	−0.0051 (8)	−0.0020 (8)	−0.0005 (8)
C4	0.0475 (10)	0.0421 (10)	0.0405 (9)	−0.0010 (8)	−0.0026 (8)	−0.0021 (8)
C5	0.0438 (10)	0.0384 (9)	0.0344 (9)	0.0058 (8)	−0.0010 (7)	−0.0005 (7)
C6	0.0385 (9)	0.0433 (10)	0.0391 (9)	0.0041 (7)	−0.0016 (7)	−0.0009 (7)
C7	0.0893 (19)	0.0635 (14)	0.0419 (11)	−0.0171 (13)	0.0195 (11)	−0.0072 (10)
C8	0.0520 (11)	0.0442 (10)	0.0514 (12)	0.0021 (9)	−0.0063 (9)	−0.0048 (9)
C9	0.0555 (13)	0.0612 (14)	0.0745 (15)	−0.0125 (11)	−0.0003 (12)	−0.0112 (12)
Cl1	0.0474 (4)	0.0764 (5)	0.1170 (6)	0.0117 (3)	−0.0114 (3)	−0.0108 (4)
N1	0.0470 (11)	0.0823 (15)	0.0683 (13)	−0.0106 (10)	0.0095 (9)	0.0072 (11)
N2	0.0367 (8)	0.0438 (8)	0.0333 (7)	0.0019 (6)	−0.0005 (6)	−0.0020 (6)
N3	0.0494 (10)	0.0628 (11)	0.0375 (8)	−0.0066 (8)	0.0062 (7)	−0.0066 (8)
N4	0.0462 (9)	0.0611 (11)	0.0423 (9)	−0.0072 (8)	0.0063 (7)	−0.0067 (8)
O1	0.1052 (15)	0.0692 (11)	0.0490 (9)	−0.0220 (10)	0.0015 (9)	−0.0161 (8)
S1	0.0431 (3)	0.0575 (4)	0.0653 (4)	−0.0022 (2)	−0.0006 (2)	0.0173 (2)

### Geometric parameters (Å, °)

**Table:**

C1—N1	1.283 (3)	C5—C7	1.488 (3)
C1—Cl1	1.707 (2)	C6—N4	1.362 (2)
C1—S1	1.720 (2)	C6—C8	1.469 (3)
C2—C3	1.340 (3)	C7—H7A	0.9600
C2—N1	1.371 (3)	C7—H7B	0.9600
C2—H2	0.9300	C7—H7C	0.9600
C3—C4	1.495 (3)	C8—O1	1.212 (3)
C3—S1	1.720 (2)	C8—C9	1.490 (3)
C4—N2	1.466 (2)	C9—H9A	0.9600
C4—H4A	0.9700	C9—H9B	0.9600
C4—H4B	0.9700	C9—H9C	0.9600
C5—N2	1.341 (2)	N2—N3	1.357 (2)
C5—C6	1.378 (3)	N3—N4	1.295 (3)
			
N1—C1—Cl1	122.58 (17)	C5—C7—H7B	109.5
N1—C1—S1	116.39 (17)	H7A—C7—H7B	109.5
Cl1—C1—S1	121.03 (15)	C5—C7—H7C	109.5
C3—C2—N1	116.9 (2)	H7A—C7—H7C	109.5
C3—C2—H2	121.6	H7B—C7—H7C	109.5
N1—C2—H2	121.6	O1—C8—C6	120.2 (2)
C2—C3—C4	127.6 (2)	O1—C8—C9	121.7 (2)
C2—C3—S1	109.37 (17)	C6—C8—C9	118.14 (19)
C4—C3—S1	123.01 (14)	C8—C9—H9A	109.5
N2—C4—C3	113.00 (16)	C8—C9—H9B	109.5
N2—C4—H4A	109.0	H9A—C9—H9B	109.5
C3—C4—H4A	109.0	C8—C9—H9C	109.5
N2—C4—H4B	109.0	H9A—C9—H9C	109.5
C3—C4—H4B	109.0	H9B—C9—H9C	109.5
H4A—C4—H4B	107.8	C1—N1—C2	109.05 (19)
N2—C5—C6	103.75 (16)	C5—N2—N3	111.21 (16)
N2—C5—C7	123.72 (18)	C5—N2—C4	130.06 (16)
C6—C5—C7	132.53 (18)	N3—N2—C4	118.71 (15)
N4—C6—C5	108.88 (17)	N4—N3—N2	107.41 (15)
N4—C6—C8	122.34 (18)	N3—N4—C6	108.75 (16)
C5—C6—C8	128.73 (18)	C1—S1—C3	88.29 (11)
C5—C7—H7A	109.5		
			
N1—C2—C3—C4	−178.2 (2)	C6—C5—N2—N3	−0.2 (2)
N1—C2—C3—S1	−0.6 (3)	C7—C5—N2—N3	178.9 (2)
C2—C3—C4—N2	−130.4 (2)	C6—C5—N2—C4	178.39 (17)
S1—C3—C4—N2	52.3 (2)	C7—C5—N2—C4	−2.5 (3)
N2—C5—C6—N4	0.3 (2)	C3—C4—N2—C5	69.8 (2)
C7—C5—C6—N4	−178.7 (2)	C3—C4—N2—N3	−111.75 (19)
N2—C5—C6—C8	−177.00 (18)	C5—N2—N3—N4	0.0 (2)
C7—C5—C6—C8	4.0 (4)	C4—N2—N3—N4	−178.78 (17)
N4—C6—C8—O1	−174.8 (2)	N2—N3—N4—C6	0.2 (2)
C5—C6—C8—O1	2.2 (3)	C5—C6—N4—N3	−0.4 (2)
N4—C6—C8—C9	4.2 (3)	C8—C6—N4—N3	177.18 (18)
C5—C6—C8—C9	−178.8 (2)	N1—C1—S1—C3	−0.6 (2)
Cl1—C1—N1—C2	−179.37 (19)	Cl1—C1—S1—C3	179.14 (15)
S1—C1—N1—C2	0.4 (3)	C2—C3—S1—C1	0.65 (18)
C3—C2—N1—C1	0.2 (3)	C4—C3—S1—C1	178.39 (17)

### Hydrogen-bond geometry (Å, °)

**Table:**

*D*—H···*A*	*D*—H	H···*A*	*D*···*A*	*D*—H···*A*
C4—H4B···N3^i^	0.97	2.48	3.399 (3)	159
C4—H4A···O1^ii^	0.97	2.48	3.376 (3)	153
C7—H7C···O1^ii^	0.96	2.57	3.396 (3)	144

Symmetry codes: (i) −*x*+1, −*y*+1, −*z*; (ii) −*x*+1, *y*−1/2, −*z*+1/2.
